# Autosomal Recessive Atrial Dilated Cardiomyopathy Due to *NPPA* Mutation in a Young Patient

**DOI:** 10.3390/jcdd13010037

**Published:** 2026-01-09

**Authors:** Massimiliano Marini, Manuela Iseppi, Silvia Quintarelli, Francesca Tedoldi, Flavia Ravelli, Roberto Bonmassari, Eloisa Arbustini

**Affiliations:** 1 Department of Cardiology, Santa Chiara Hospital, 38122 Trento, Italy; 2 Laboratory of Biophysics and Translational Cardiology, Department of Cellular, Computational and Integrative Biology (CIBIO), Centre for Medical Sciences (CISMed), University of Trento, 38123 Trento, Italy; 3 Department of Research, Centre for Inherited Cardiovascular Disease, IRCCS Foundation, University Hospital Policlinico San Matteo, 27100 Pavia, Italy

**Keywords:** atrial fibrillation, atrial cardiomyopathy, genetic disorders, case report

## Abstract

**Background:** Atrial dilated cardiomyopathy (ADCM) related to homozygous *Natriuretic Peptide Precursor A* (*NPPA*) pathogenic variants is an exceptionally rare inherited atrial cardiomyopathy characterized by progressive atrial enlargement, supraventricular arrhythmias, and eventual atrial standstill. **Case summary:** We report the case of a 9-year-old girl identified through population genetic screening as a homozygous carrier of the *NPPA* c.449G>A (p.Arg150Gln) variant who subsequently developed symptomatic paroxysmal atrial fibrillation (AF) at the age of 18. Although baseline cardiac investigations were normal, her current evaluation shows biatrial enlargement with preserved ventricular function. She underwent radiofrequency pulmonary vein isolation; however, recurrent symptomatic AF persists, requiring ongoing antiarrhythmic therapy and long-term oral anticoagulation (CHA_2_DS_2_-VA: 0; HAS-BLED: 0). Notably, patients with *NPPA*-related ADCM have a markedly increased thromboembolic risk due to progressive atrial mechanical failure, and anticoagulation should therefore be considered irrespective of conventional clinical risk scores. **Discussion and conclusions:** This case highlights the importance of genetic testing in young patients with atrial fibrillation and no underlying structural heart disease. The early identification of *NPPA*-related atrial dilated cardiomyopathy may aid in risk stratification and guide rhythm and anticoagulation management. Expanding genetic screening in select individuals with isolated atrial fibrillation may facilitate earlier diagnosis in this exceptionally rare condition.

## 1. Introduction

Atrial standstill, also referred to as atrial paralysis, is an exceptionally rare myocardial disorder characterized by the complete absence of both electrical and mechanical atrial activity. It may be idiopathic or secondary to a range of conditions, including neuromuscular dystrophies, laminopathies, cardiac amyloidosis, and Ebstein’s anomaly. In the early stages, it may present with episodes of atrial fibrillation (AF) and subsequently progress to atrial standstill with an absence of atrial excitability and bradycardic junctional escape rhythm [1].

The idiopathic familial form of atrial dilated cardiomyopathy (ADCM) has been increasingly recognized and linked to specific genetic variants. In our previous paper we first reported the homozygous *Natriuretic Peptide Precursor A* (*NPPA*) c.449G>A (p.Arg150Gln) gene variant in 13 patients with ADCM and atrial standstill [2]. More recently, the same homozygous pathogenic variant was identified by Forleo et al. [3] and Silva et al. [4] in patients presenting with persistent AF and atrial cardiomyopathy.

We describe the youngest symptomatic case of homozygous *NPPA*-related ADCM reported so far, which adds meaningful insight into the early natural history of this exceedingly rare atrial cardiomyopathy. This case reinforces the importance of recognizing atrial dysfunction as a potential early manifestation of inherited cardiomyopathy and highlights the value of incorporating atrial abnormalities into heart failure screening strategies.

## 2. Case Presentation

**Figure 1:**
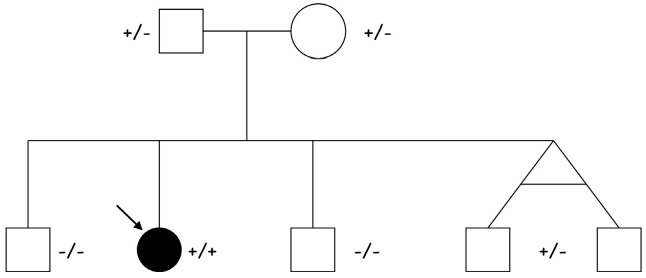
Pedigree of the proband (indicated by the arrow) affected by ADCM due to a homozygous [c.449G>A (p.Arg150Gln)] *NPPA* mutation. Both parents (I:1 and I:2) and two siblings, homozygous twins, (II:4 and II.5) are heterozygous healthy carriers, while two siblings (II:1 and II:3) are healthy non-carriers.

Following the identification of the homozygous *NPPA* variant in our previous familial study [2], we extended genetic screening to the surrounding area in northeastern Italy, enrolling 583 individuals. This population was derived from a small mountain community where an endemic familial form of atrial standstill had already been documented, with eight affected cases reported [5]. During this screening program, a homozygous c.449G>A (p.Arg150Gln) mutation was detected in an asymptomatic 9-year-old girl. She was referred to our cardiology unit, where baseline investigations including ECG, two-dimensional transthoracic echocardiography, cardiac MRI, and Holter monitoring were all within normal limits. The parents were clinically healthy heterozygous carriers of the *NPPA* gene variant. Among the proband’s four clinically unaffected male siblings, two were confirmed to be heterozygous carriers of the identified pathogenic variant (Figure 1).

**Figure 2:**
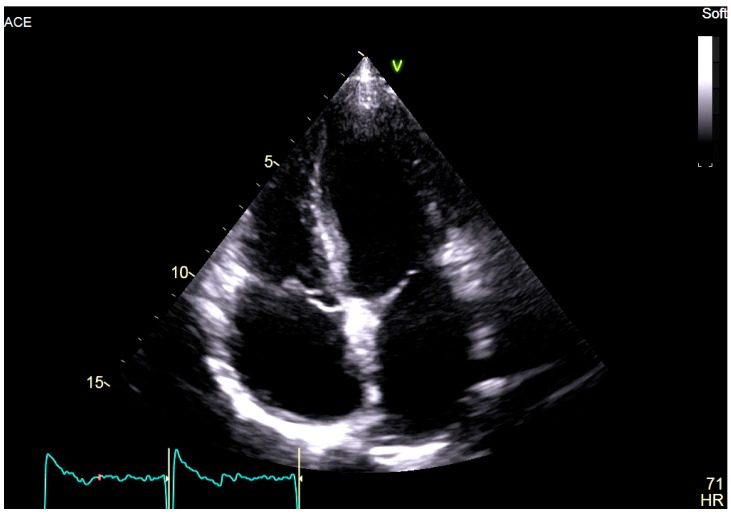
Two-dimensional transthoracic echocardiography, apical 4-chamber view of the proband, showing marked biatrial enlargement with preserved biventricular size and systolic function.

At the age of 18, the patient complained of palpitations during physical activity, and an episode of atrial flutter after a gastrointestinal infection was also documented. An implantable loop recorder was subsequently implanted for the purpose of defining the burden of AF and detecting asymptomatic episodes. More recent echocardiography revealed biatrial dilatation (left atrium diameter: 38 mm; left atrium volume index: 39 mL/m^2^; right atrium area: 21 cm^2^), mild mitral regurgitation, and preserved biventricular function (left ventricular ejection fraction: 58%) (Figure 2).

**Figure 3:**
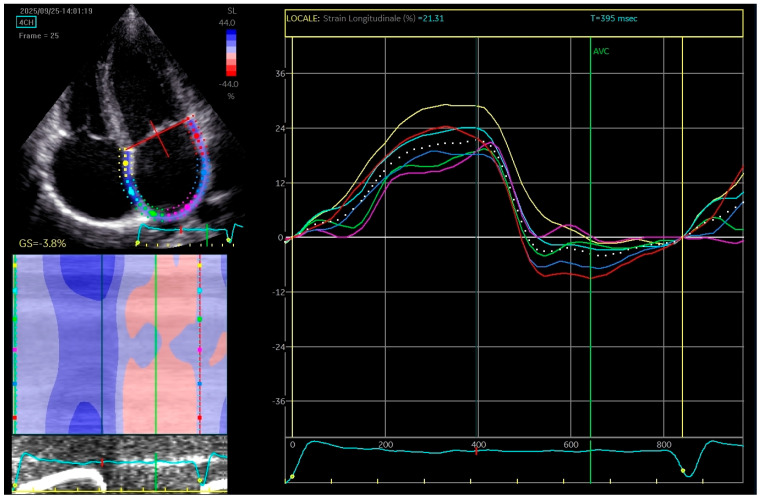
The apical four-chamber view was utilized for the strain measurements of the left atrium (LA); the edge of the LA endocardium was manually traced. The software generated tracings based on the 2D strain of LA. The mean deformation (strain) is expressed in percentage and calculated by the software.

Two-dimensional speckle tracking echocardiography was conducted, revealing a mild reduction in left atrial function based on strain and strain rate parameters, which indicates increased stiffness of the atrium (Figure 3).

**Figure 4:**
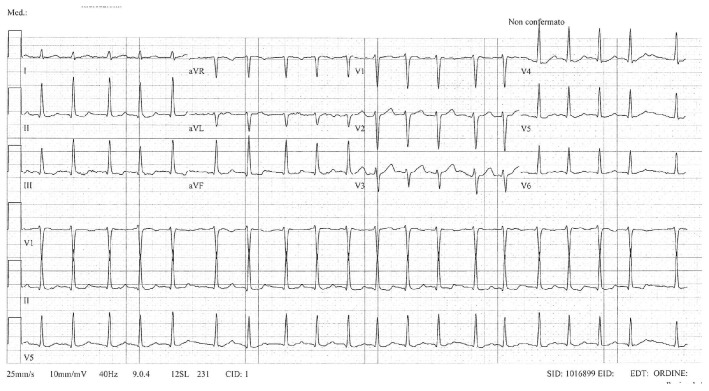
ECG showing atrial fibrillation.

Due to recurrent symptomatic episodes of atrial fibrillation (AF) (Figure 4) refractory to antiarrhythmic therapy (flecainide 150 mg/day), she underwent an electrophysiological study, which revealed a low-voltage area of the anterior and posterior wall of the left atrium. Pulmonary vein isolation using radiofrequency (RF) ablation was performed, along with a posterior wall line, achieving left atrial box isolation.

**Figure 5:**
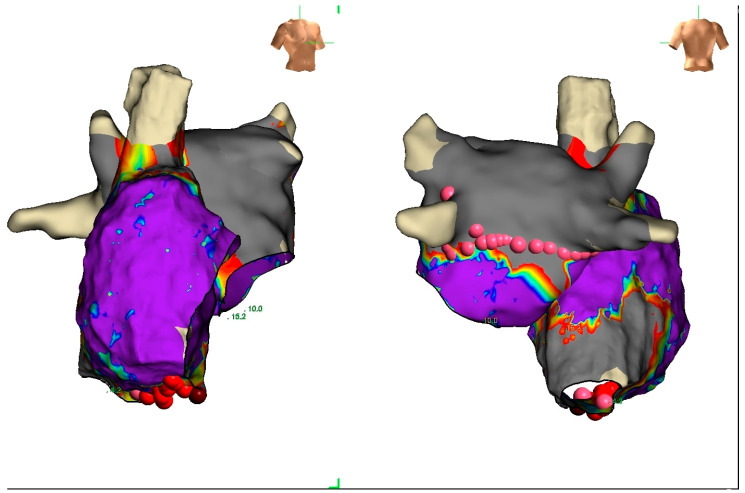
The figure shows an anterior and posterior view of a voltage biatrial electroanatomic map (voltage range between 0.05 mV and 0.5 mV). The right atrium is larger than the left with a limited area of fibrosis of the lateral wall.

Electroanatomic mapping documented biatrial enlargement but not atrial paralysis or inexcitability (Figure 5) and the atrioventricular node conduction was within normal limits.

Despite the ablation therapy, the patient continues to experience episodes of atrial fibrillation and is therefore still under antiarrhythmic prophylaxis (Flecainide 150 mg/die); she is also receiving anticoagulation therapy (CHA_2_DS_2_-VA: 0; HAS-BLED: 0).

## 3. Discussion

The occurrence of atrial fibrillation in young patients without structural cardiac abnormalities is unusual and merits thorough investigation to determine the underlying etiology. In our previous paper [2], we delineated the natural progression of idiopathic atrial dilatation culminating in atrial standstill and identified a homozygous *NPPA* missense variant (p.Arg150Gln) in all affected individuals from six unrelated families. As a missense mutation, this variant leads to an amino acid substitution in the atrial natriuretic peptide precursor, potentially altering peptide structure and biologic function and contributing to progressive atrial remodeling. The disease phenotype was characterized by adult-onset presentation, pronounced biatrial enlargement, early supraventricular arrhythmias, progressive electrical atrial inexcitability, thromboembolic complications, and preserved left ventricular systolic function [2,6]. The clinical course of this patient is consistent with the pathophysiology of ADCM, which typically begins with supraventricular tachyarrhythmias due to the progressive atrial electrical inactivity and fibrosis replacement. However, in this young female, the clinical evolution appears to be unusually premature, and she represents the youngest symptomatic patient of our case series. It is conceivable that additional secondary mutations may be present, potentially contributing to an earlier onset of the disease. To date, including the present case, sixteen homozygous carriers of the *NPPA* p.Arg150Gln variant have been reported in the literature [2,3,4], including both males and females (nine females and seven males). This slight difference does not allow for meaningful epidemiological conclusions, and no sex-related difference in prevalence or penetrance has emerged, which is consistent with its autosomal recessive inheritance.

Furthermore, the therapeutic role of catheter ablation, specifically pulmonary vein isolation and posterior line with RF ablation, is highly questionable in this context and lacks support in the current literature. Similarly, the decision to initiate anticoagulation, particularly with the use of direct oral anticoagulants, remains debatable and was not employed in our previous patients treated with a vitamin K antagonist. In *NPPA*-related atrial dilated cardiomyopathy, the thromboembolic risk appears to be largely driven by the progressive loss of atrial contractile function, which may ultimately result in atrial standstill. Under these circumstances, the absence of effective atrial emptying favors blood stasis independently of traditional clinical risk factors. Thromboembolic events, including ischemic stroke, have already been documented in affected homozygous individuals and may even represent the first clinical manifestation [2]. Therefore, standard risk stratification tools such as the CHA_2_DS_2_-Va score may underestimate the actual thrombotic burden in this specific condition, supporting the rationale for long-term anticoagulation even in young patients with low clinical scores.

Our findings highlight the relevance of expanding genetic screening in young individuals presenting with atrial fibrillation and no underline disease, as it may personalize therapeutic decision-making.

## 4. Conclusions

Atrial dilated cardiomyopathy (ADCM) associated with the homozygous *NPPA* p.Arg150Gln variant constitutes an exceptionally rare clinical entity, marked by severe atrial enlargement, atrial standstill, bradycardic junctional escape rhythm, and an elevated risk of thromboembolic events. We report the youngest documented case of ADCM caused by the homozygous pathogenic *NPPA* variant, identified through population screening and presented clinically with atrial fibrillation. Further investigation is warranted to elucidate the molecular mechanisms underlying *NPPA*-related atrial pathology and to assess the therapeutic potential of interventions targeting natriuretic peptide signaling pathways. The implementation of early genetic screening in carefully selected at-risk populations may facilitate prompt diagnosis, enable pre-emptive management, and potentially mitigate the progression to heart failure.

## Data Availability

The raw data supporting the conclusions of this article will be made available by the authors on request.

## Abbreviations

The following abbreviations are used in this manuscript:

NPPA	Natriuretic Peptide Precursor A
AF	Atrial Fibrillation
ADCM	Atrial dilated cardiomyopathy
ECG	Electrocardiogram
